# KindMap: an e-mental health tool to promote the well-being and mental health of people facing infertility—study protocol for a feasibility randomised control trial

**DOI:** 10.1136/bmjopen-2024-087447

**Published:** 2024-12-09

**Authors:** Naír Carolino, Marina Cunha, José Pinto-Gouveia, Sofia Gameiro, Ana Galhardo

**Affiliations:** 1 Instituto Superior Miguel Torga, Coimbra, Portugal; 2 CINEICC—Faculty of Psychology and Educational Sciences, University of Coimbra, Coimbra, Portugal; 3 School of Psychology, Cardiff University, Cardiff, UK

**Keywords:** MENTAL HEALTH, eHealth, Feasibility Studies, Psychosocial Intervention, REPRODUCTIVE MEDICINE, Self-Help Devices

## Abstract

**Introduction:**

Fertility patients increasingly use web-based and mobile-based apps to access psychosocial care. These digital tools may be a helpful alternative to traditional psychological interventions. Developing and evaluating patient-centred e-mental health tools rooted in evidence-based interventions is a priority. The KindMap is a stand-alone, cost-free e-mental health intervention derived from adapting the Mindfulness Based Programme for Infertility (MBPI) contents to a digital format. The KindMap integrates mindfulness and self-compassion skills training and Acceptance and Commitment Therapy components. This protocol is intended to evaluate the KindMap’s feasibility and explore the extent to which the web-app mode of delivery limited efficacy results are similar to the MBPI in-person format results. Furthermore, it will test the causal theory underlying KindMap.

**Methods and analysis:**

A two-arm 2:1 non-blinded feasibility randomised controlled trial (RCT) will be conducted. Participants are people dealing with infertility, who are able to access the Internet and understand Portuguese or English. Consent participants will complete an online survey at 3-time assessment moments. After baseline assessment, participants will be randomised into the KindMap experimental group (KindMap-EG; with immediate access to the web app) or the waiting-list control group. The primary outcome is well-being (WHO Index-5); secondary outcomes are infertility-related stress (Fertility Problem Inventory—Short Form), anxiety and depression (Patient Health Questionnaire for Depression and Anxiety-4); mindfulness (Five Facet Mindfulness Questionnaire—Short Form), self-compassion (Self-Compassion Scale—Short Form), psychological flexibility (Psy-Flex) and infertility-related self-efficacy are the potential mechanisms of change. KindMap-EG will also complete a feasibility survey.

**Ethics and dissemination:**

The study was approved by the Ethics Committee of the Faculty of Psychology and Educational Sciences of the University of Coimbra (Identifier: CEDI/FPCEUC:78/R_10). The KindMap study may contribute to the existing research on e-health technologies applied to mental health. The study outcomes will be disseminated through publications in peer-reviewed journals and national and international conference presentations.

**Trial registration number:**

NCT05899374.

**STRENGTHS AND LIMITATIONS OF THIS STUDY**

- The KindMap is a web app developed as a stand-alone, cost-free e-mental health intervention that entails a conceptual replication of a previously tested Mindfulness Based Programme for Infertility.
- The study is a two-arm, parallel-group, non-blinded feasibility study with 2:1 computer-generated randomised allocation to the KindMap experimental group or waiting-list control group.
- The study has a longitudinal design and addresses the following feasibility dimensions: demand, adaptation, acceptability, implementation, practicality, integration and limited efficacy.
- Being a feasibility study, the small sample size will not allow for determining the intervention’s efficacy, but will inform the design of a future suitably powered randomised controlled trial.

## Introduction

 Infertility is a clinical condition defined as a reproductive system dysfunction related to the inability to achieve a natural pregnancy after 12 months or more of regular and unprotected sexual intercourse.^1^ The WHO estimates that 8%–12% of reproductive-aged couples worldwide are affected by this condition.^2^ The emotional and psychological impact of infertility is well documented, encompassing profound emotional, relational and social consequences that can lead to long-term mental health impairments.^3 4^ People facing an infertility diagnosis who are pursuing medical treatment tend to face additional burdens due to self-injections, medical examinations, oocyte retrieval, embryo transfer and the (potential) experience of failed treatment cycles. This journey can trigger stress, anxiety and depressive symptoms,^5^,^8^ as well as maladaptive coping mechanisms (eg, experiential avoidance and self-judgement),^9 10^ ultimately diminishing overall quality of life.^11^

Psychological interventions directed at people with infertility have proven effective in improving mental health outcomes.^12^,^16^ Cognitive-behavioural therapies tailored to individuals dealing with infertility have been shown to enhance emotion regulation skills and alleviate psychopathological distress, enhancing well-being.^17^,^22^ One of these interventions designed for people with infertility is the Mindfulness Based Programme for Infertility (MBPI).^19^ The MBPI is an in-person group intervention comprising psychoeducation, mindfulness, compassion and Acceptance and Commitment Therapy (ACT) components. The MBPI was an effective intervention, with results showing a significant decrease in psychopathological symptoms and a significant improvement in emotion regulation processes (eg, mindfulness skills and self-efficacy to deal with infertility) compared with the control group.^19^ Furthermore, participants in the MBPI group continued to reveal therapeutic benefits at a 6-month follow-up and experienced lasting results for up to 7 years.^23^

Fertility patients increasingly use web-based and mobile-based apps to access psychosocial care because these overcome identified barriers, such as financial constraints, travel, scheduling issues, apprehension, fear of being denied/excluded from treatment and stigmatisation.^24 25^ These e-mental health tools are becoming widespread and may be, to some extent, a helpful alternative to traditional psychological interventions, aligning with patients’ needs and preferences.^26^

Therefore, developing and evaluating patient-focused e-mental health tools rooted in evidence-based interventions should be a priority.^3^ Although several support apps have been developed,^27 28^ very few have been evaluated and almost none using high-quality randomised controlled trials (RCTs).^27 29 30^ An exception was MyJourney, a self-guided psychosocial intervention targeting a somehow different group, people who did not fulfil their wish for a child. The MyJourney app has shown to be feasible, revealing high demand, good acceptability and adaptation, and preliminary evidence of efficacy.^31^

In this context, a web app, KindMap, was developed as a stand-alone, cost-free e-mental health intervention derived from adapting MBPI contents to a digital format. The KindMap integrates mindfulness and self-compassion skills training and ACT components. Mindfulness-based programmes, including self-compassion components, are effective in reducing psychopathological distress and have shown a ‘trans-diagnostic effect’ across different clinical conditions, being a highly recommended approach for people coping with infertility.^20^ ACT has also been related to better mental health outcomes in individuals dealing with health conditions (Gloster *et al*, for a review of meta-analyses).^32^ However, studies focusing on ACT within the infertility field are still limited.^33^ Since infertility is a significant life stressor, ACT emerges as a potentially suitable approach that could yield positive and protective effects among individuals experiencing infertility. ACT may contribute to improving mental health indicators, such as anxiety, depression, stress and quality of life, through its targeted components. Furthermore, a systematic review and meta-analysis conducted by Klimczak *et al* also found that ACT could be effectively delivered as a self-guided online intervention.^34^ To our knowledge, none of the developed support apps targeting people with infertility integrates ACT components.^27 29 31^ KindMap will be the first e-mental health integrating mindfulness, self-compassion and ACT components specifically designed for people dealing with infertility.

### Objectives

The current study was designed to evaluate KindMap’s feasibility, specifically feasibility dimensions, such as demand, adaptation, acceptability, implementation, practicality, integration and limited efficacy.^35^ Feasibility results will allow answering to whether the online stand-alone format will be as acceptable as its in-person format and whether one can expect preliminary efficacy of the therapeutic activities incorporated in the intervention. Moreover, it will be possible to assess the extent to which people will sustain their engagement with the tool.

Results will be reported according to the Consolidated Standards of Reporting Trials guidelines for feasibility and pilot studies^36^ and will enlighten needed improvements and adjustments to be performed in the KindMap app and study protocol. If feasible, this study will help define methodological aspects to proceed to the KindMap efficacy study through a large RCT.

## Methods and analysis

### Study design

A two-arm, parallel-group, non-blinded feasibility study with 2:1 computer-generated randomised allocation (www.random.org) to the KindMap experimental group (KindMap-EG; prompt access to KindMap web app) or waiting-list control group (WL-CG; access to KindMap after 10 weeks). The study will encompass three assessment moments: baseline (T1; pre-intervention), 8–10 weeks after baseline (T2; post-intervention) and 10 weeks after post-intervention (T3; only for KindMap-EG). Although there is a recommendation for completing a module per week (similar to the MBPI sessions), the KindMap may be used at the participant’s own pace. Therefore, T2 will occur considering a 2-week flexible period. A T3 assessment will also be implemented at a 10-week interval.

### Participants and recruitment

Recruitment will take place between June and December 2024. An informative email and social media accounts containing information about the KindMap study were created and will be disseminated through the Portuguese Fertility Association (APFertilidade; Patients’ Association) and Fertility Europe (an umbrella organisation of European patients’ associations involved with infertility issues). Interested people will be guided to the KindMap’s landing page, where they can sign up to participate in the study.

Inclusion criteria: (a) presenting self-reporting an infertility diagnosis, (b) age between 18 and 45, (c) being at any stage of the fertility journey (eg, waiting for test results, actively involved in medical treatment, on a wait-list for treatment, having completed one or more treatment cycles), (d) having Internet access and (e) understanding Portuguese or English. Exclusion criteria: (a) currently undergoing any form of psychological intervention, (b) being pregnant and (c) being unable to use the KindMap due to other health difficulties (eg, hearing or visual impairments), all self-reported.

### Procedures

On registering on the KindMap landing page, participants will be asked to complete a questionnaire addressing the eligibility criteria. Participants who meet the eligibility criteria will receive an email containing detailed information about the study and a link to an online platform where they will be asked to provide informed consent (using several mandatory checkboxes) and then proceed to complete the T1 assessment. After T1, consenting participants will be randomly allocated to the KindMap-EG or WL-CG. KindMap-EG participants will have immediate access to the KindMap web app; 8–10 weeks after the baseline, all participants will be invited to complete the T2 assessment by email. At this moment, participants in the WL-CG will be given access to the KindMap web app. 10 weeks later (T3), participants in the KindMap-EG will be invited to complete the T3 assessment. Participants not completing assessments (T1, T2 or T3) will receive reminder emails on days 3, 6 and 9. After receiving the reminders, participants who have not completed the assessment will receive a brief exit questionnaire inquiring about the reasons for withdrawal. The assessment protocols will be hosted on Limesurvey.

### Sample size

G*Power calculations for within-between interaction Analysis of Variance (ANOVA) repeated measures (two-conditions, two-time points), assuming a p=0.05, effect size *f*=0.25, power=0.95, recommend a sample size of 54. Assuming participation and retention rates of 60% and 34% (observed in similar apps), we will recruit n=180 participants. This sample size is in accordance with Sim and Lewis^37^ recommendation of at least 50 participants for feasibility studies.

### Randomisation

**Figure 1:**
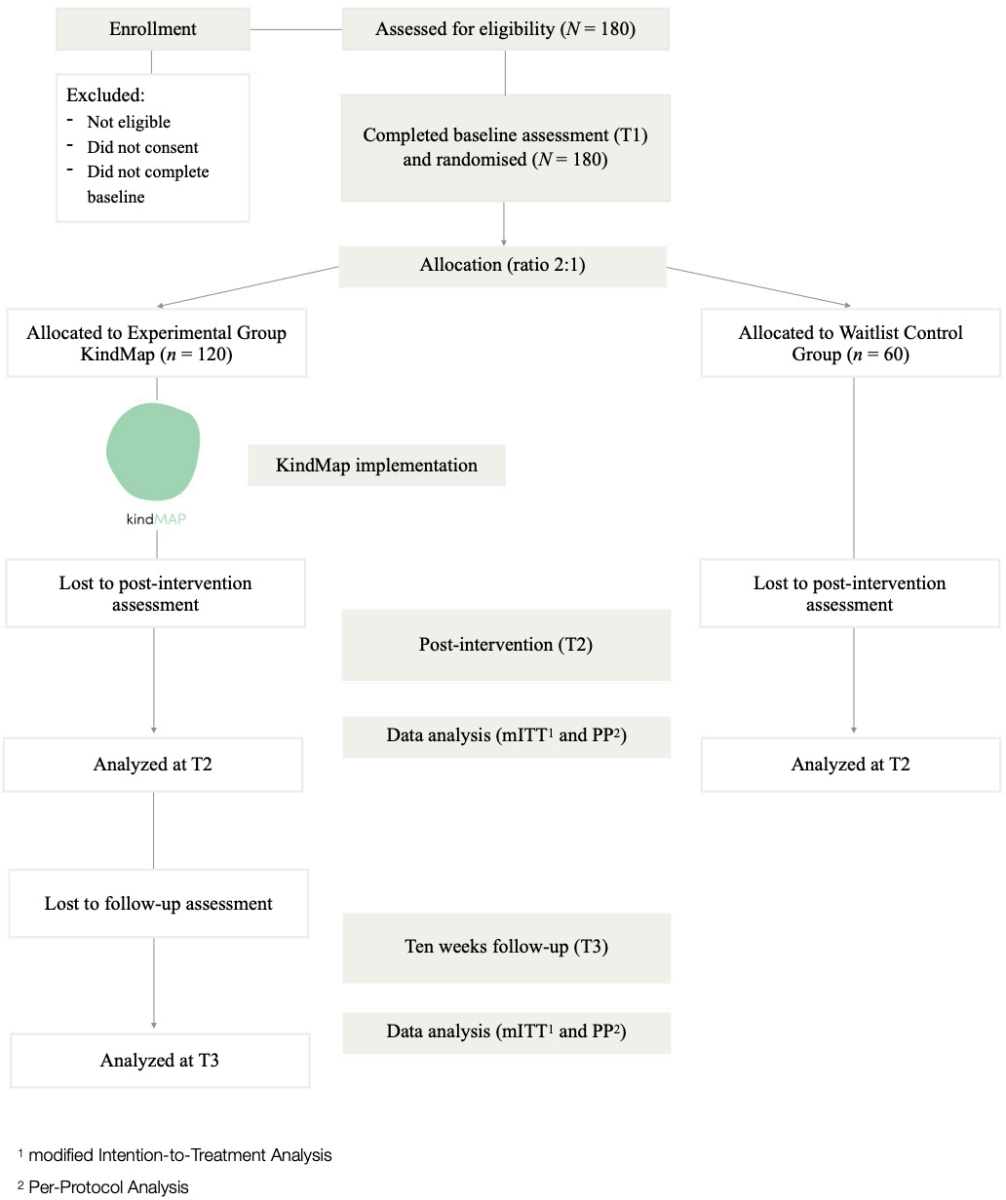
Participant flowchart.

Figure 1 shows the expected flow of participants throughout the study. Consenting participants will be randomised in a 2:1 ratio through computer-generated randomisation after completing the T1 assessment. Randomisation will be guaranteed by a third researcher (different from the two responsible for enrolment and allocation of the participants to the EG-KindMap and WL-CG groups) who will only know the participants’ code. Participants randomised into KindMap-EG will be stratified into a Portuguese or English-speaking group. All participants will be informed of the randomisation results.

### Intervention

The KindMap is a stand-alone, cost-free e-mental health tool presenting a low-intensive psychological intervention to be used at users’ own time and pace. The KindMap intervention is described using the Template for Intervention Description and Replication.^38^

The KindMap web app was developed by a multidisciplinary research team following the framework commissioned by the Medical Research Council and the National Institute of Health Research for developing complex interventions^39 40^ and a User-Centred Design methodology. This intervention was derived from adapting the MBPI’s content to a web app format. The MBPI contents were developed based on the Contextual Cognitive-Behavioural Therapies (CCBT) framework, incorporating mindfulness, self-compassion and ACT components.

The KindMap will be available in European Portuguese and English at www.kindmap.pt. KindMap’s landing page will provide the background and aims of the intervention, the target audience, a summary of the contents of its eight modules and a brief presentation of the KindMap team. As a stand-alone tool, the KindMap can be simultaneously and freely accessed by any number of interested people and used at users’ convenience time and pace. For the duration of the feasibility study, only participants meeting eligible criteria will have access to the KindMap. It is a completely self-guided web app, with no human support, but a support contact list will be provided. For technical problems, an email address will be available.

**Figure 2:**
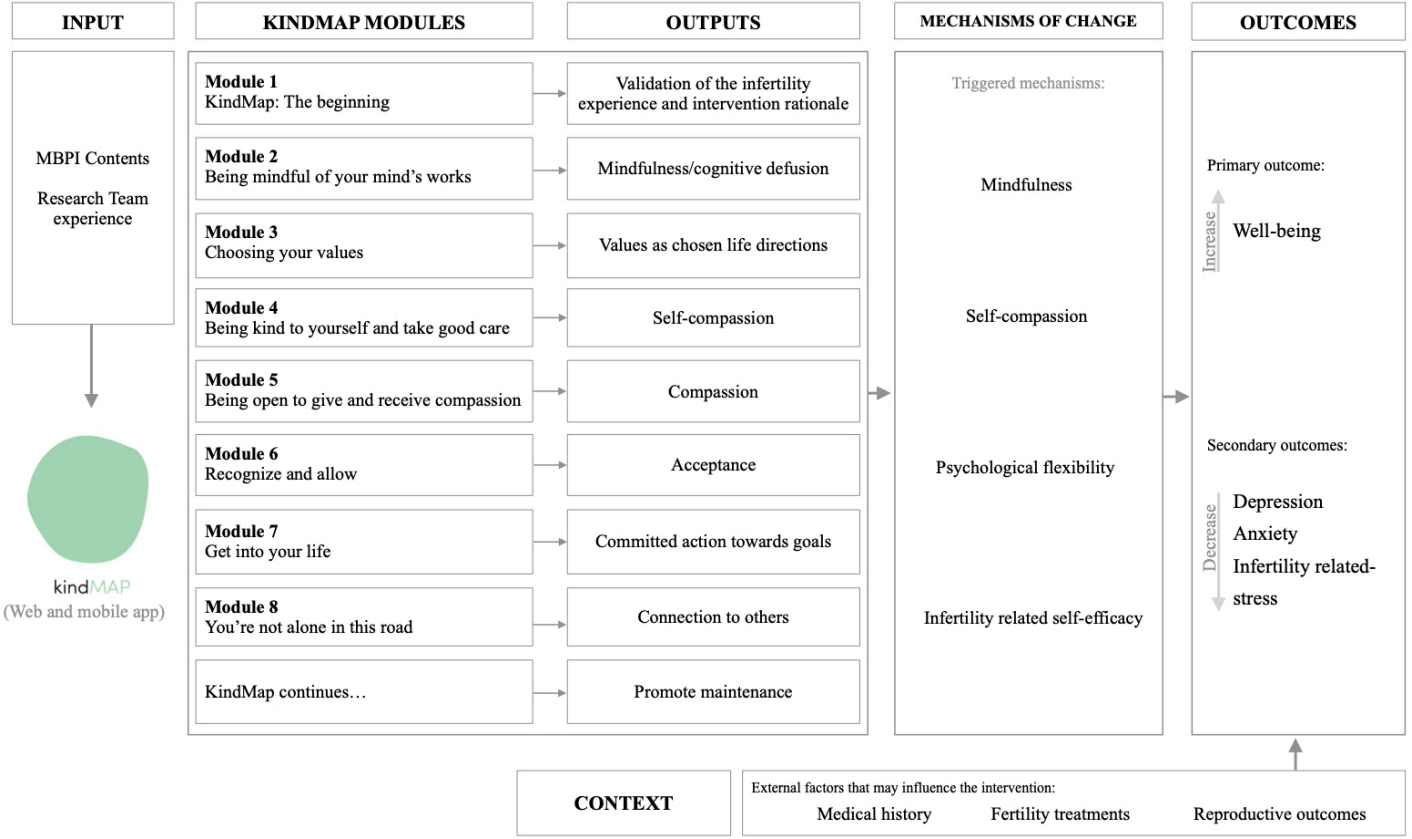
KindMap logic model.

**Figure 3:**
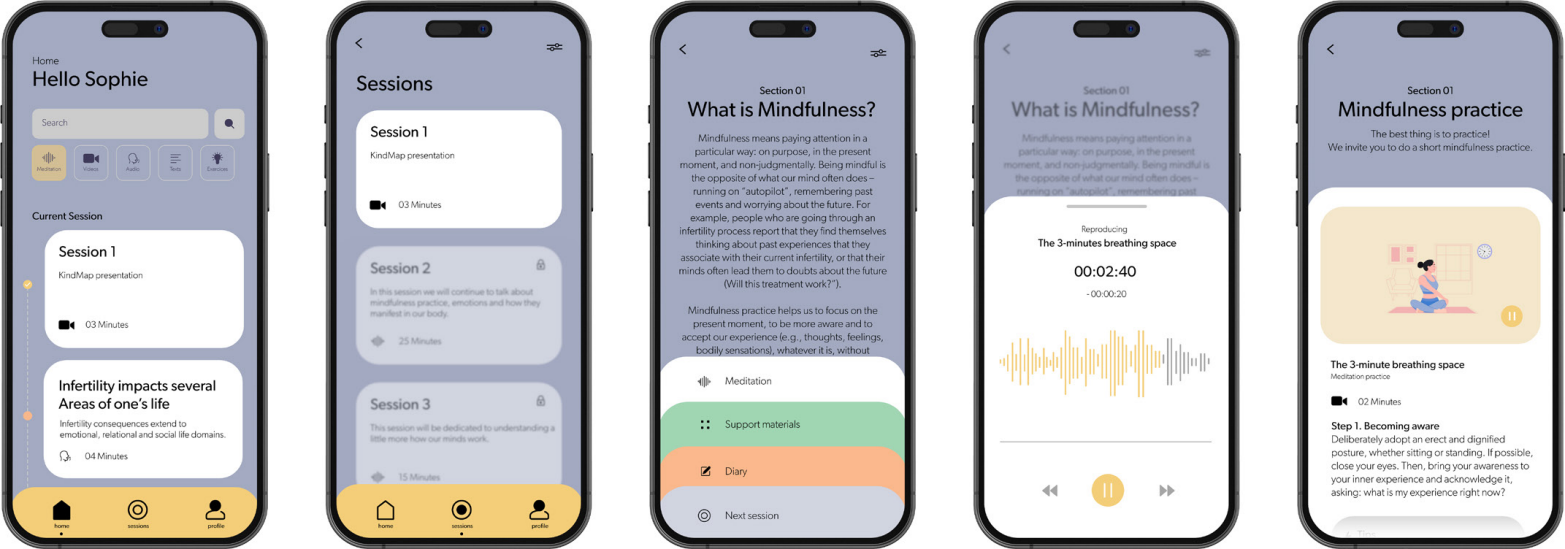
KindMap screenshots of the mobile app.

The KindMap comprises eight modules addressing psychoeducation, mindfulness and compassion-guided practices, and ACT-based experiential exercises (table 1). Each module is structured to heighten understanding of a specific subject and cultivate a particular therapeutic skill (figure 2). The modules’ contents are delivered through text, video, audio, experiential exercises, prompts for reflection and interactive content to promote users’ engagement (figure 3). The recommendation is to address a module per week, leading to 8 weeks of using the KindMap.

**Table 1:** KindMap modules, contents, practices and exercises

Module		Contents	Practices and exercises
1	KindMap: The Beginning	Life areas affected by infertilityThe psychological impact of infertilityThe concept of mindfulnessDifficulties in the mindfulness practiceMindfulness practice instructions	*Meditation practice—The three-minute breathing spaceMeditation practice—Being in the present moment*
2	Being mindful of your mind’s work	Being in the present momentHow the mind and emotions workSimple ways of being present	*Experiential exercise—Being in the present momentMeditation practice—Body Scan*
3	Choosing your values	How the mind worksPassengers on the bus metaphorValues clarification	*Meditation practice—Sounds and thoughts meditationExperiential exercise—Values identification/clarification*
4	Being kind to yourself and taking good care	Lifestyle considerations—eating, physical exercise, alcohol, nicotine, sleep, relationships,Self-careSelf-compassion	*Experiential exercise—The time pieMeditation practice—Compassion mindfulnessExperiential exercise—Compassionate postcardMeditation practice—Moment of self-compassion*
5	Being open to give and receive compassion	Interpersonal relationshipsDealing with other people’s commentsThe impact of infertility on the coupleCompassion for others and receiving compassion from others	*Meditation practice—Giving and taking meditationExperiential exercise—Dyad communication*
6	Recognise and allow	Be an explorer on the expedition of your mindAcceptanceThe pain in my headTo live the experience as it is	*Meditation practice—Leaves on a streamMeditation practice—Exploring difficultyExperiential exercise—The pain in my head*
7	Get into your life	Dealing with challenges and obstaclesGoose in the bottle enigmaCommitted action	*Experiential exercise—Obstacles in the riverChallenge—Goose in the bottle enigmaMeditative practice—Being where you are*
8	You’re not alone in this road	Facing the challenge of infertilityPeople dealing with infertility testimonies	*Meditative practice—Brief loving-kindnessMeditative practice—Mindfulness: being present*

Note: Every KindMap module starts with a brief theme introduction and ends with a gentle reminder quote and a module summary. At the end of each module, practices and exercises are suggested to be completed until the next module.

After completing each module, users unlock the following module and have automatic access to the ‘Practices’ section activities/meditations to practice between modules. The final topic addressed is a wrap-up of the programme designed to encourage users to recognise the changes they have undergone since the first module, acknowledge the therapeutic skills they have acquired/developed and motivate them to incorporate these skills in their everyday lives and upcoming difficult situations.

Every time users access the web app, they are invited to answer two brief questions about their well-being state and state of mindfulness (present moment awareness), using a 10-point Likert scale ranging from *not at all* (1) to *very/strongly* (10).

There will be no special criteria for discontinuing or modifying allocated interventions.

### Measures

At baseline (T1), sociodemographic and clinical information will be collected (eg, gender, age, marital status, infertility type and cause, previous fertility treatments and former psychological counselling) combined with standardised self-report measures of well-being, infertility-related stress, depression and anxiety. These self-report measures will also be completed at T2 and T3. Additionally, at T2, the feasibility questionnaire regarding the KindMap intervention and the study protocol will be completed by the KindMap-EG participants.

#### Feasibility outcomes

Aligned with Bowen *et al*,^35^ the current study uses several feasibility dimensions to evaluate the KindMap intervention and study protocol. For the KindMap intervention, demand, adaptation, acceptability, implementation, practicality, integration and limited efficacy dimensions will be assessed.

Demand will be assessed by programme adherence (eg, number of users and percentage of modules completed). Adaptation will be assessed by the differences in the hours spent and total number of visits of participants engaging with the KindMap in Portuguese and English. Acceptability will be assessed by user-reported programme satisfaction, perceived usefulness of each module and meditation practices and willingness to recommend the KindMap to peers. Implementation will be assessed by responses to open-ended questions regarding technical problems, the suitability of the 8-week recommended engagement period and barriers and facilitators of use. Practicality will be assessed by the number of participants who use the KindMap as intended (complete eight modules), complete six out of eight modules during the 8–10-week recommended engagement period, and the time taken to use the web app as intended. Integration will be assessed by participants’ perception of using the KindMap skills in everyday life and their willingness to continue doing so. Limited efficacy will be assessed by the effect size estimations of primary and secondary self-reported outcomes changes in the EG-KindMap versus WL-CG. More specifically, Modified Intention-to-Treat (mITT, all participants randomised) and Per-Protocol (PP, only participants who received a sufficient dose) analyses on primary (well-being) and secondary outcomes (infertility-related stress, depression and anxiety) measured at T1, T2 and T3 will be performed.

For the study protocol, feasibility dimensions of demand, acceptability, implementation, practicality and adaptation will be evaluated. Demand will be assessed by participation and retention rates and reasons for non-participation/withdrawal; acceptability will be addressed by the proportion of participants who completed T1, T2 and T3 assessment moments; implementation by the reported issues concerning study procedures or materials; practicality by the time taken to complete assessments and adaptation by the participation and attrition rates according to the chosen language version of the KindMap. A traffic-light progression criteria will be used for each feasibility outcome.^41^

#### Primary outcome

As the primary outcome, well-being will be assessed using the WHO Index-5 (WHO-5).^42^ WHO-5 is a five-item self-report questionnaire of current well-being. It is a unidimensional measure with responses rated on a 6-point Likert scale, ranging from *at no time* (0) to *all the time* (5). Lower scores indicate the worst possible quality of life, and higher scores indicate the best possible quality of life.

#### Secondary outcomes

As secondary outcomes, infertility-related stress, depression and anxiety will be assessed. The Fertility Problem Inventory—Short Form (FPI-SF)^43^,^45^ is a 27-item self-report instrument designed to assess perceived infertility stress based on a comprehensive approach. FPI-SF includes four domains: social concern, need for parenthood, rejection of a child-free lifestyle and couple’s relationship concern. The social concern domain involves being sensitive to comments, reminders of infertility, experiencing feelings of social isolation and feeling disconnected from family or peers (eg, ‘When I see families with children, I feel left out’). The need for parenthood domain is connected to viewing parenthood as a fundamental or essential life goal and strongly identifying with the role of being a parent (eg, ‘As long as I can remember, I’ve wanted to be a parent’). Rejection of a child-free lifestyle domain refers to the belief that future satisfaction or happiness is contingent on having a child, accompanied by a negative perspective on a lifestyle without children (eg, ‘Not having a child/another child would allow me time to do other satisfying things’). The couple’s relationship concerns domain involves challenges in discussing infertility, worries about how infertility might affect the relationship and concerns about its impact on the couple’s sexual life (eg, ‘My partner doesn’t understand the way the fertility problem affects me’). The FPI-SF is rated on a 6-point Likert scale, ranging from *strongly disagree* (1) to *strongly agree* (6).

The Patient Health Questionnaire for Depression and Anxiety (PHQ-4)^46 47^ is a 4-item self-report instrument widely used to identify individuals who may be suffering from anxiety, depression or both. It includes two subscales: the depression subscale and the anxiety subscale. The depression subscale comprises the two core criteria for depressive disorders (eg, ‘Feeling down, depressed or hopeless’). The anxiety subscale encompasses the two core criteria for generalised anxiety disorder (eg, ‘Feeling nervous, anxious or on edge’), which have also been proven to be effective screening items for panic disorder, social anxiety and post-traumatic stress disorder. The PHQ-4 is rated on a 4-point Likert scale, ranging from *not at all* (0) to *nearly every day* (3). Higher scores are strongly associated with functional impairment and disability.

#### Mechanisms of change

Mindfulness, self-compassion, psychological flexibility and infertility-related self-efficacy will be assessed as mechanisms of change.

The Five Facet Mindfulness Questionnaire—Short Form (FFMQ-SF)^48^,^50^ is a 24‐item self‐report questionnaire highly sensitive to change, aiming to assess different mindfulness skills. The FFMQ-SF is rated on a 5-point Likert scale and comprises five subscales: observing (eg, ‘I pay attention to how my emotions affect my thoughts and behaviour’), describing (eg, ‘I can easily put my beliefs, opinions and expectations into words’), acting with awareness (eg, ‘When I do things, my mind wanders off, and I’m easily distracted’), non-judging of inner experience (eg, ‘I tell myself I shouldn’t be feeling the way I’m feeling’) and non-reactivity to inner experience (eg, ‘I perceive my feelings and emotions without having to react to them’). Higher scores are indicative of higher levels of mindfulness facets.

The Self-Compassion Scale—Short Form^51^,^53^ is a 12-item self-report measure used to assess the ability to deal with and embrace one’s feelings of suffering based on a sense of warmth, connection and concern. This instrument is widely used in clinical practice for its high sensitivity to change. It integrates two subscales: Self-Disparagement (eg, ‘When I fail at something important to me, I become consumed by feelings of inadequacy.’) and Self-Care (eg, ‘I try to be understanding and patient towards those aspects of my personality I don’t like.’). The total score is an overall suggestion of self-compassion, with higher scores linked to more self-compassion.

The Psy-Flex^54 55^ is a context-sensitive self-report measure aiming to assess psychological flexibility, defined as the competencies that guide the individual to change and facilitate behaviours that are more adaptive and valued by the individual, following the ACT theoretical model. This instrument includes six items, rated on a 5-point Likert scale, each referring to one of the central skills when developing psychological flexibility. Higher scores are indicative of higher levels of psychological flexibility.

The Infertility Self-Efficacy Scale (ISE)^56 57^ is a self-report instrument to assess infertile patients' perception of their ability to use their own cognitive, emotional and behavioural competencies for dealing with infertility diagnosis and medical treatments. ISE comprises 16 items rated on a 9-point Likert scale. A higher total score indicates higher confidence levels in perceived capabilities to deal with infertility and its demands.

### Data analysis plan

Statistical analysis will be conducted using IBM SPSS Statistics V.27. Descriptive statistics will be performed. Specifically, for the continuous variables, means and SD or SE of the mean will be obtained, and for categorical variables, absolute numbers and percentages (%) will be calculated. Differences between groups will be assessed through t*-*tests and χ^2^ tests. mITT (all participants who will complete T1 and T2) and PP (only participants who will receive a sufficient dose) analyses on primary (well-being) and secondary outcomes (infertility-related stress, depression and anxiety) measured at T1, T2 and T3 will be performed to explore limited efficacy on the study outcomes.

### Patient and public involvement statement

This manuscript is a protocol outlining the process of KindMap’s feasibility assessment. There was no patient or public involvement in defining the research questions or study design. The participation burden was considered when selecting the outcome instruments and developing the feasibility questionnaire. A group of reproductive healthcare professionals and potential users (fertility patients) will be included in the following study’s stage, and their KindMap experiences will be explored as part of the feasibility assessment of the intervention. Study findings will be available to enrolled participants and key project stakeholders.

### Ethics and dissemination

The KindMap’s study is registered at ClinicalTrials.gov (Identifier: NCT05899374, date assigned 05/04/2023). All ethical and deontological requirements inherent to scientific research and intervention in Psychology and Medical Sciences will be attended to, namely the guidelines for studies with human beings (Declaration of Helsinki (1964), European Legislation, Portuguese Psychologists Association). The study was approved by the Ethics Committee of the Faculty of Psychology and Educational Sciences of the University of Coimbra (Identifier: CEDI/FPCEUC:78 /R_10). Data will be stored securely and confidentially, following General Data Protection Regulation guidelines. Results will be published in peer-reviewed journals and national and international conference presentations.

## Discussion

The KindMap is an e-mental health tool developed to address the increasing demand for online cost-free and evidence-based support for people negatively impacted by infertility. The KindMap will be the first digital intervention to integrate psychoeducation, mindfulness, compassion and ACT components. Besides being based on a previously evaluated programme, the KindMap was developed based on users’ preferences and needs (eg, attractiveness, design, clarity), as well as newly delivered content formats (text, video, audio, experiential exercises and interactive content), and its feasibility remains unknown. Considering the novelty of the KindMap and the limited number of feasibility and efficacy studies conducted in e-mental health tools tailored to people dealing with infertility, this feasibility study is relevant in informing intervention refinements and procedures for a future randomised controlled trial.^36^

The study defined in this protocol will allow us to assess specific feasibility dimensions using traffic-light progression criteria and to explore whether it is worth conducting a future RCT to evaluate the efficacy of KindMap, a strongly recommended procedure.^35 36^ It is expected that KindMap reveals to be a feasible low-intensive psychological intervention with limited efficacy results pointing to improvements in well-being and in the perception of self-efficacy to deal with infertility, as well as in mental health indicators (infertility-related stress, anxiety and depression) when compared with a wait-list control group in whom no changes are expected. These preliminary efficacy results may help establish a more comprehensive theoretical framework for the therapeutic change processes of CCBT targeting people dealing with infertility by clarifying the link between changes in emotion regulation processes and changes in well-being and mental health indicators.

Finally, the KindMap study may contribute to the existing research on e-health technologies applied to mental health, being aligned with the European^58^ and Portuguese^59^ agenda for digital healthcare transition, fostering the integration of patient-centred web-based technologies into clinical practices and improving the sustainability and efficacy of health systems.

The KindMap web app can reach a broader number of fertility patients, being disseminated through fertility clinics and patients’ associations, providing additional support aligned with a more patient-centred care framework. The KindMap may also impact clinical practice and services. It may be used as a stand-alone tool and as a supplement to psychological interventions delivered by mental health professionals.

## supplementary material

10.1136/bmjopen-2024-087447 online supplemental file 1
