# Supplementary material for: KindMap: an e-mental health tool to promote the well-being and mental health of people facing infertility—study protocol for a feasibility randomised control trial
Source: BMJ Open. 2024 Dec 9;14(12):e087447. doi: 10.1136/bmjopen-2024-087447 (PMC11628952; doi:10.1136/bmjopen-2024-087447)
Supplement: online supplemental file 1 [file bmjopen-14-12-s001.docx]

**KINDMAP'S FEASIBILITY STUDY**

We would like to invite you to participate in a research project designed to explore the feasibility of an *e-mental health* tool for people facing fertility problems, the KindMap. The KindMap was derived from a previously developed psychological intervention program – the Mindfulness Based Program Intervention (MBPI) – tested in a face-to-face group format, revealing sustained results regarding mental health indicators. The current study is being conducted by a research team from CINEICC - Faculty of Psychology and Educational Sciences at the University of Coimbra.

To explore how people use **KindMap** and its effects on their well-being and mental health indicators, we would like to understand different types of experiences and uses. So, if you decide to participate, you will be randomly allocated to one of two groups: one group will have immediate access to the KindMap, and another will have access to it eight weeks later.

Participants who have immediate access to KindMap will be asked to complete three online assessment moments: initially (just before having access to KindMap), eight weeks later, and three months after using KindMap.

Participants who only have access to KindMap eight weeks later will be asked to fill in two online assessment moments: initially and eight weeks later. These participants will have access to KindMap even if they choose not to complete the second online assessment moment.

Participants will be asked to complete a set of self-report questionnaires addressing demographic data, well-being, mental health indicators, and potential mechanisms of change. A questionnaire assessing the participants' experience of using KindMap will be used. The collected data will also be used to explore which KindMap's components might be related to changes in the outcomes.

Each assessment moment will take no longer than 20 minutes to complete. Your participation in completing the questionnaires at each moment is very important to us, regardless of whether you have been given access to KindMap, how you have chosen to use it, and how satisfied you are.

When you indicate your interest in participating in the study and give us your consent, you will be asked to provide your email address (which will be used to send you the links to fill in the questionnaires and, possibly, gentle reminders). You will be asked to generate an alphanumeric code to pair your answers at the different assessment moments.

Your data will be stored confidentially in accordance with GDPR regulations. Only the researchers on this project will be able to access your data. Your data will be anonymised one month after the survey has closed, and after this point, no-one will be able to trace your information back to you. You can ask for the information you have provided to be deleted at any time up until the data has been anonymised, and you can have access to the information up until the data has been anonymised.

**What benefits can there be if you participate in the KindMap study?**

**KindMap** is an adaptation of the Mindfulness Based Programme for Infertility (MBPI), which is based on Contextual Cognitive Behavioural Therapies and has shown beneficial effects on mental health and psychological adjustment in people with infertility. These effects were maintained for seven years, regardless of whether the participants had children or not. In addition, research has shown that participating in this type of study can help participants better understand their thoughts and emotions.

**Are there any disadvantages and/or risks to participating in the KindMap study?**

Some of the exercises suggested by KindMap may make you more emotional by involving thinking and writing about your infertility experience (something that will only be accessible to you).

If you have any problems or questions about the study and the participation, please contact [kindmapstudy@gmail.com](mailto:kindmapstudy@gmail.com)

**Informed Consent**

- I understand that my participation in this research project will involve being randomly selected for a group that will use the KindMap web app as they wish for a period of eight weeks and answer an online questionnaire before, immediately after, and three months after using it, or for a group that will answer an online questionnaire at two different time points, spaced eight weeks, after which I will have access to the KindMap web-app to use as I wish.
- I understand that participation in the study is completely voluntary and that I can withdraw at any time without giving reasons.
- I understand that I am free to ask any questions at any time. I am free to clarify or discuss my doubts with the research team.
- I understand that some of the KindMap modules may make me reflect on my personal experience and make me more emotional, and I am free to withdraw at any time.
- I understand that I may receive reminders by email at each of the assessment moments and while I am using KindMap.
- I understand that personal data will be processed in accordance with the regulations of the National Data Protection Commission.
- I understand that I will receive additional information and feedback about the study results at the end of the project.

**Do you agree to participate in the study?**

- **Yes** (by answering yes, you declare that you are aware of the study's aims and the involvement you are being asked to participate in. You declare that you are participating voluntarily. You also agree that the data will be analysed anonymously and aggregated by the researchers within the scope of the study's aims).
- **No, I am not interested in participating in the study.**
