# Supplementary material for: KindMap: an e-mental health tool to promote the well-being and mental health of people facing infertility—study protocol for a feasibility randomised control trial
Source: BMJ Open. 2024 Dec 9;14(12):e087447. doi: 10.1136/bmjopen-2024-087447 (PMC11628952; doi:10.1136/bmjopen-2024-087447)
Supplement: Supplementary file 2 [file bmjopen-2024-087447.reviewer_comments.pdf]

PEER REVIEW HISTORY

BMJ Open publishes all reviews undertaken for accepted manuscripts. Reviewers are asked to complete a checklist review form and are provided with free text boxes to elaborate on their assessment. These free text comments are reproduced below.

ARTICLE DETAILS

Title (Provisional)

KindMap - an e-mental health tool to promote the well-being and mental health of people facing infertility: Study protocol for a feasibility randomized control trial

Authors

Carolino, Naír; Cunha, Marina; Pinto-Gouveia, José; Gameiro, Sofia; Galhardo, Ana

---

VERSION 1 - REVIEW

**Reviewer**	**1**
**Name**	**Tsampras, Nikolaos**
**Affiliation**	**Manchester University Foundation Trust**
**Date**	**06-Jul-2024**
**COI**	**No conflict of interest**

This is a protocol for a feasibility study, aiming to assess the efficacy of a mental health support app for subfertile patients.

The protocol is well written and the objectives are clear.The methodology is well described.

I recommend approval of the protocol

**Reviewer**	**2**
**Name**	**Faramarzi, Mahbobeh**
**Affiliation**	**Babol University of Medical Science**
**Date**	**13-Jul-2024**
**COI**	**None**

A good protocol in the field of infertility. A few things need to be clarified:

In the introduction, it was explained that in choosing the type of psychological support, the two treatments of calm mind and commitment have been merged together.

In the method section, the type of intervention should be explained. Also, the intervention modules should be explained in a table.

Determine how adherence to treatment is measured in KindMap

VERSION 1 - AUTHOR RESPONSE

Reviewer: 1

Dr. Nikolaos Tsampras, Manchester University Foundation Trust

Comments to the Author:

This is a protocol for a feasibility study, aiming to assess the efficacy of a mental health support app for subfertile patients. The protocol is well written and the objectives are clear. The methodology is well described. I recommend approval of the protocol.

The authors thank the reviewer for the thoughtful words.

Reviewer: 2

Dr. Mahbobeh Faramarzi, Babol University of Medical Science

Comments to the Author:

A good protocol in the field of infertility.

The authors thank the reviewer for the helpful suggestions.

A few things need to be clarified:

In the introduction, it was explained that in choosing the type of psychological support, the two treatments of calm mind and commitment have been merged together.

The authors are unsure about the comment concerning the two treatments mentioned by the Reviewer. There is no treatment approach called calm mind in the KindMap intervention. We believe the Reviewer's reference to "commitment" relates to Acceptance and Commitment Therapy (ACT). In fact, there are ACT components that were included in the KindMap intervention (e.g., ACT-based experiential exercises). As suggested, a table with the KindMap components was added to the manuscript, which may clarify these components (p. 9, line 244).

In the method section, the type of intervention should be explained. Also, the intervention modules should be explained in a table.

The KindMap intervention is already described in the manuscript, in the Methods section, with the subtitle "Intervention". Furthermore, as suggested, we included a table allowing for a more global understanding of the intervention (p. 9, line 244).

Determine how adherence to treatment is measured in KindMap

Adherence to the treatment is a component of the demand dimension. This is mentioned in the Methods section, in the subtitle Measures – Feasibility outcomes: "Demand will be assessed by program adherence (e.g., number of users and percentage of modules completed)."
